# Effect of antiplatelet therapy on cardiovascular and kidney outcomes in patients with chronic kidney disease: a systematic review and meta-analysis

**DOI:** 10.1186/s12882-019-1499-3

**Published:** 2019-08-07

**Authors:** Xiaole Su, Bingjuan Yan, Lihua Wang, Jicheng Lv, Hong Cheng, Yipu Chen

**Affiliations:** 1 Division of Nephrology, Beijing Anzhen Hospital, Capital Medical University, No. 2, Anzhen Street, Chaoyang District, Beijing, China; 2 Division of Nephrology, Shanxi Medical University Second Hospital, Shanxi Kidney Disease Institute, No.382, Wuyi Road, Xinghualing Distirct, Taiyuan, China; 3 Division of Nephrology, Peking University First Hospital, Peking University Institute of Nephrology, No.8, Xishiku Street, Xicheng District, Beijing, China

**Keywords:** Antiplatelet therapy, Chronic kidney disease, Cardiovascular events, Meta-analysis

## Abstract

**Background:**

The benefits and risks of antiplatelet therapy for patients with chronic kidney disease (CKD) remain controversial. We undertook a systematic review and meta-analysis to investigate the effects of antiplatelet therapy on major clinical outcomes.

**Methods:**

We systematically searched MEDLINE, Embase, and the Cochrane Library for trials published before April 2019 without language restriction. We included rrandomized controlled trials that involved adults with CKD and compared antiplatelet agents with controls.

**Results:**

Fifty eligible trials that included at least one event were identified, providing data for 27773patients with CKD, including 4518 major cardiovascular events and 1962 all-cause deaths. Antiplatelet therapy produced a 15% (OR, 0.85; 95% CI 0.74–0.94) reduction in the odds of major cardiovascular events (*P* = 0.002), a 48% reduction for access failure events (OR, 0.52; 95% CI, 0.31–0.73), but had no significantly effect on all-cause death (OR, 0.87; 95% CI, 0.71–1.01) or kidney failure events (OR, 0.87; 95% CI, 0.32–1.55). Adverse events were significantly increased by antiplatelet therapy, including major (OR, 1.33; 95% CI, 1.11–1.59) or minor bleeding (OR, 1.66; 95% CI, 1.27–2.05). Among every 1000 persons with CKD treated with antiplatelet therapy for 12 months, 23 major cardiovascular events will be prevented while nine major bleeding events will occur.

**Conclusions:**

Major prevention with antiplatelet agents (cardiovascular events and access failure), might outweigh the risk of bleeding, and there seemed to be an overall net benefit. Individual evaluation and careful monitoring are required.

**Electronic supplementary material:**

The online version of this article (10.1186/s12882-019-1499-3) contains supplementary material, which is available to authorized users.

## Background

Chronic kidney disease (CKD) is recognized as a major public health problem [1]. Cardiovascular disease (CVD) is the leading cause of morbidity and mortality for CKD patients [2]. The association between prevalent CVD and the risk of progression of CKD has been demonstrated by large population-based cohort studies [2–5]. Management of multiple cardiovascular risk factors, such as improved blood pressure and diabetes control, not only protects from cardiovascular disease, but also delays CKD progression [6]. In patients with occlusive vascular disease, antiplatelet therapy reduced the yearly risk of major cardiovascular events, including myocardial infarction, stroke, and vascular death, by about 25%. The different etiological pathways of CVD, pathophysiology, and abnormal platelet function have resulted in substantial uncertainty concerning the risks and benefits of antiplatelet therapy in patients with CKD [7, 8].

In addition to providing cardiovascular protection, another role of antiplatelet therapy is presumed to prevent dialysis vascular access thrombosis and improve fistula or graft function in populations on or nearing commencement of hemodialysis therapy [9]. Some random control trials (RCTs) involving a CKD population showed that more intensive platelets inhibition could be of reduced benefit in preventing major cardiovascular events [10, 11] and dialysis vascular access failure [12], whereas others suggested benefits of similar or even greater magnitude [13–15]. Given these uncertainties, patients with CKD have been shown to be less likely to be prescribed antiplatelet drugs, even after acute myocardial infarction [16, 17]. Thus, the issue of whether these beneficial effects could be outweighed by the increased bleeding hazards remains inconclusive and controversial. It is difficult for clinicians to interpret these results when counseling patients with CKD about antiplatelet therapy.

In this systematic review, our aim was to summarize all the available clinical trial data and evaluate the benefits and side effects of antiplatelet therapy in preventing major cardiovascular events, all-deaths, dialysis vascular access failure, and kidney outcome in patients with CKD.

## Methods

### Data sources and searches

We conducted a systematic review based on standard methods, including a pre-specified protocol registered at PROSPERO [18] (CRD42016037842) and reporting in accordance with the PRISMA guidelines [19]. The following databases were searched without language restriction before April 2019: MEDLINE by the Ovid, Embase, and the Cochrane Library database using relevant keywords and medical subject headings that included all spellings of known RCTs, CKD, and antiplatelet agents (see Additional file 1 for full search terms). Reference lists from identified trials and review articles and the ClinicalTrials.gov website were scanned manually to identify any other relevant studies.

### Study selection and outcome estimation

We included data from RCTs in which any antiplatelet agent was given to patients with CKD compared with placebo or usual therapy. Studies were eligible for inclusion if they were included adults with CKD. Antiplatelet drugs included the broad categories: cyclooxygenase-2 inhibitor, adenosine diphosphate P2Y12 receptor inhibitor, thromboxane A2 synthase and receptor inhibitor, platelet glycoprotein IIb/IIIa receptor blockade and phosphodiesterase inhibitor.

Predefined outcomes that contained analyzable data were listed as follows:

First, major cardiovascular events, defined as a composite, including fatal or non-fatal myocardial infarction, fatal or non-fatal stroke, coronary artery revascularization, and cardiovascular death. When myocardial infarction, stroke, and cardiovascular death were reported concurrently in one trial, myocardial infarction and stroke were extracted if they reported no-fatal data. Participants could have suffered more than one type of non-fatal event. Second, all-cause death. Third, kidney failure events, including more than 25% or 50% decrease in estimated glomerular filtration rate (eGFR), doubling of serum creatinine, or end-stage renal disease (ESRD). Fourth, dialysis vascular access failure, including early thrombosis, loss of unassisted patency, failure to attain suitability for dialysis, and need for access intervention. Fifth, adverse events, consisting of major bleeding (fatal, life-threatening, disabling, requiring hospital admission, or comparable definitions used by individual authors) or minor bleeding (all other reported bleeding events). Sixth, the rate of change in eGFR per year. Positive differences represented a slower decline in the treatment group than in the control group. Last, changes in serum creatinine and proteinuria from baseline to the end of follow-up. Negative differences represented a greater decrease in the treatment group than in the control group.

### Data extraction and quality assessment

Data were extracted independently by 2 authors (X.S. and B.Y.), and disagreements were resolved via consultation with the third investigator (Y.C.). A standardized form was used to extract the following data: study design, patient characteristics, renal function, type and dose of antiplatelet drugs, change in serum creatinine, the eGFR, and proteinuria or albuminuria, outcome events and adverse events.

The methodological quality of each included study was assessed independently by 2 authors using the Cochrane Collaboration risk-of-bias tool [20] according to the developed criteria with the eight validity domains (Additional file 2), in which an assessment of financial conflicts of interest was included [21]. The Jadad scale was also used to quantify the study quality [22].

### Statistical analysis

The results were expressed as odds ratios (ORs) with 95% confidence intervals (CI) for binary outcomes. A random-effects model using a fully Bayesian method was applied, which assumes a binomial likelihood on the log-odds scale for a binary outcome [23, 24]. The non-informative priors with vague normal (mean, 0; variance, 100,000) and uniform (0–1) prior distributions for parameters was used. We generated 55,000 simulations for each of the two sets of different initial values, and we discarded the first 5000 simulations as the burn-in period (see codes in Additional file 3). The achievement of convergence was assessed using the Brooks-Gelman-Rubin statistic [25]. According to the predefined protocol [18], different statistical methods, including DerSimonian-Laird [26], empirical Bayes and restricted maximum likelihood [27] estimators with the CIs constructed Knapp-Hartung approach [28], were also used in sensitivity analysis.

The change of proteinuria or albuminuria was calculated using the standardized mean differences, and the change of serum creatinine and the rate of change in eGFR per year were pooled using mean differences. The rate of change in eGFR means the difference from the baseline eGFR divided by the number of years between creatinine measurements. A random effects model was used to pool mean differences and standardized mean differences.

*I*^*2*^ and *tau*^*2*^ statistics were used to estimate the heterogeneity. We did several sensitivity analyses to explore potential reasons for heterogeneity or inconsistency [18]. Those planned in advance were exclusion of: studies with sample sizes less than 200; studies with follow-up years less than 1 year; studies with Jadad scores less than 3. Pre-specified subgroup analysis was performed to investigate the source of heterogeneity by several major covariates, including stage of CKD, number of patients, mean age, type and doses of antiplatelet drugs, and follow-up duration [18]. Post-hoc subgroup analysis was conducted based on different participants. Chi-squared test and meta-regression were both used to assess the heterogeneity between subgroups [29]. We summarized the quality of evidence using GRADEprofiler version 3.6.1 according to the Grading of Recommendations Assessment, Development, and Evaluation guidelines (GRADE) [30]. We assessed potential publication bias with Egger’s tests and the visual interpretation of funnel plots. A two-sided *P*-value less than 0.05 was considered statistically significant. Metafor packages from R software version 3.1.1, WinBUGs version 1.4.3, and STATA version 12.0 were used for the statistical analyses.

## Results

**Fig. 1:**
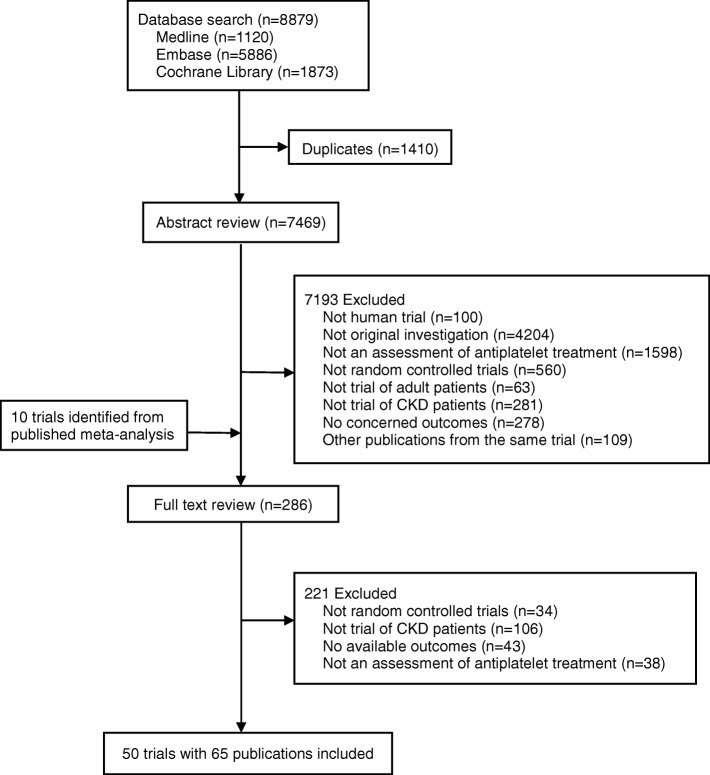
Process for the identification of eligible studies

We identified 8879 records. After removal of duplicates, we screened the abstracts and selected 286 publications for full-text review, including 10 trails with unpublished data of CKD patients identified from published meta-analyses [30–32]. A total of 50 eligible trials reported in 65 publications with 27,773 participants were included in this review (Fig. 1) [10–13, 15, 33–93].

We summarized the characteristics of the included studies in Additional file 4: Table S1 and Additional file 5: Table S2. Median follow-up duration was 12 months (range 1–90 months). Forty trials were placebo controlled and ten trials were usual-care controlled. Overall, we studied six types of antiplatelet medicines, including cyclooxygenase-2 inhibitor for 12 studies, phosphodiesterase inhibitor for five studies, adenosine diphosphate P2Y12 receptor inhibitor for 15 studies, platelet glycoprotein IIb/IIIa receptor blockade for seven studies, thromboxane A2 synthase receptor inhibitor for three studies, and combined therapy for eight studies. The risk of bias varied substantially across the studies. Twenty-eight trials had a Jadad scale of 4 or 5, and others were scored ≤3. The results from the Cochrane Collaboration risk-of-bias tool are shown in Additional file 6: Figure S1 and Additional file 7: Figure S2.

### Effect of antiplatelet therapy on cardiovascular outcomes and death

**Fig. 2:**
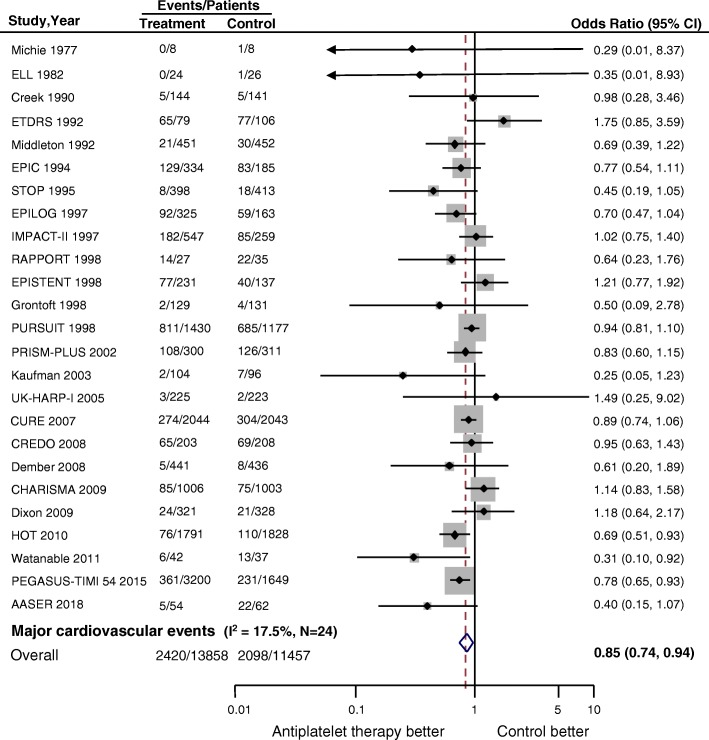
Forest plot for major cardiovascular events. Major cardiovascular events were defined as a composite, including fatal or non-fatal myocardial infarction, fatal or non-fatal stroke, coronary artery revascularization, and cardiovascular death. *CI* confidence interval, *N* number of trials

Data regarding the effects of antiplatelet therapy on major cardiovascular events were available from 25 trials, which included 25,315 participants and 4518 events. Overall, compared with placebo or usual-care control groups, antiplatelet therapy produced a 15% reduction in the odds of cardiovascular events (OR, 0.85; 95%CI 0.74–0.94; *P* = 0.002; Fig. 2), without evidence of heterogeneity in the results of individual trials (*I*^2^ = 17.5%, *P* for heterogeneity = 0.2).

**Fig. 3:**
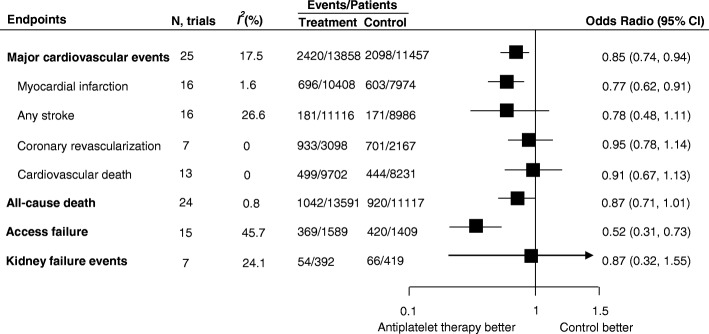
Summary of the odds radios of all outcomes. Major cardiovascular events were defined as a composite, including fatal or non-fatal myocardial infarction, fatal or non-fatal stroke, coronary artery revascularization, and cardiovascular death. Kidney failure events were defined as more than 25% or 50% decrease in eGFR, doubling of serum creatinine, or end-stage renal disease (ESRD). *N* number of trials

As a component of major cardiovascular events, fatal or nonfatal myocardial infarction events were reported in 16 trials, which included 18,382 patients and 1299 events. Antiplatelet therapy reduced the odds of myocardial infarction by 23% (OR, 0.77; 95% CI, 0.62–0.91; Fig. 3), without evidence of heterogeneity (*I*^*2*^ = 1.6%; *P* for heterogeneity = 0.4) compared with placebo or usual-care control groups. Sixteen trials reported strokes, including hemorrhagic stroke (six trials 6044 patients and 47 events) and presumed ischemic stroke (ten trials, 14,058 patients and 305 events). Overall, there was no effect of antiplatelet therapy on the risks of any stroke (OR, 0.78; 95% CI, 0.48–1.11; Fig. 3), without significant evidence of heterogeneity between trials (*I*^*2*^ = 26.6%; *P* for heterogeneity = 0.2). The risk of coronary artery revascularization was also not altered by antiplatelet therapy (seven studies, 5265 participants and 1634 events; OR, 0.95; 95% CI, 0.78–1.14; Fig. 3), without evidence of significant heterogeneity (*I*^*2*^ = 0%).

Cardiovascular death was reported in 13 trials, including 17,933 patients and 943 events. Twenty-four trials reported all-cause deaths, including 24,708 patients and 1962 events. Our analysis showed no clear effect of antiplatelet therapy on the risk of cardiovascular death (OR, 0.91; 95% CI, 0.67–1.13; *I*^*2*^ = 0%) or all-cause death (OR, 0.87; 95% CI, 0.71–1.01; *I*^*2*^ = 0.8%) compared with placebo or usual-care control groups, with CIs that were compatible with modest effects in either direction (Fig. 3 and Additional file 8: Figure S3).

There was no statistical heterogeneity for major cardiovascular events and deaths in the subgroup analyses according to prespecified characteristics (Additional file 9: Table S3).

### Effect of antiplatelet therapy on kidney outcomes

Only seven trials, including 811 participants and 140 events, provided the data for kidney failure events. There was no clear evidence that antiplatelet therapy reduced the odds of kidney failure events (OR, 0.87; 95% CI, 0.32–1.55; Fig. 3), without significant evidence of heterogeneity (*I*^*2*^ = 24.1%, *P* for heterogeneity = 0.1). The effects of antiplatelet therapy on the rate of change in eGFR and change in serum creatinine were available in seven trials with 3934 participants, and four trials with 144 participants, respectively. For studies that were placebo or usual-care controlled, antiplatelet therapy did not slow the rate of eGFR decline (MD, 0.15 mL/1.73 m^2^/year; 95% CI, − 0.89 to 1.20; *I*^*2*^ = 40.8%, *P* for heterogeneity = 0.1; Additional file 10: Figure S4) or the change in serum creatinine (MD, − 7.92 μmol/L; 95% CI − 30.41 to 14.56; *I*^*2*^ = 84.7%, *P* for heterogeneity < 0.001; Additional file 11: Figure S5). Eight studies with a total of 367 participants provided data for proteinuria. The standardized mean difference in change in proteinuria or albuminuria was significant at − 0.90 (95% CI, − 1.34 to − 0.47, *P* < 0.001) compared with the placebo or usual-care control groups, with significant heterogeneity (*I*^*2*^ = 70%, *P* for heterogeneity = 0.001; Additional file 12: Figure S6). The smaller sample size and fewer trials meant that most of subgroup analyses for kidney outcomes according to the prespecified characteristics could not be performed (Additional file 9: Table S3).

### Effect of antiplatelet therapy on hemodialysis vascular access

Data were available for 789 events of dialysis vascular access failure in 15 trials (2998 patients). When compared with placebo or usual-care control groups, antiplatelet treatment produced an apparent beneficial effect for access failure events (OR, 0.52; 95% CI, 0.31–0.73) with moderate heterogeneity (*I*^*2*^ = 45.7%, *P* for heterogeneity = 0.03; Additional file 13: Figure S7). Subgroup analysis showed that there was heterogeneity for the effects of different types of drugs (Additional file 9: Table S3).

### Effects on adverse events (major and minor bleeding)

Thirty trials (26,118 participants) reported 2707 bleeding events, including 884 major bleeding events in 27 trials and 1821 minor bleeding events in 23 trials. Overall, antiplatelet therapy induced a significant increase in the odds of bleeding (OR, 1.55; 95% CI, 1.25–1.84), with clear heterogeneity (*I*^*2*^ = 35.2%; *P* for heterogeneity = 0.03; Additional file 14: Figure S8) compared with placebo or usual-care control groups. Of these, the odds of major bleeding events were increased by 33% (OR, 1.33; 95% CI, 1.11–1.59), without evidence of heterogeneity (*I*^*2*^ = 0%), and minor bleeding by 66% (OR, 1.66; 95% CI, 1.27–2.05), with moderate heterogeneity (*I*^*2*^ = 48%, *P* for heterogeneity = 0.03). There was no statistical heterogeneity in the subgroup analyses according to prespecified characteristics (Additional file 9: Table S3).

### Net absolute effect

We calculated the absolute risk reduction per 1000 patients with CKD and NNTs (number needed to be treated, i.e. the number of patients who must be treated to prevent one adverse event) for significant benefited outcome from antiplatelet therapy (Table 1). Overall, for every 1000 people with CKD treated with antiplatelet therapy for 12 months (median follow-up duration), 23 patients (95% CI, 9–39) will avoid a major cardiovascular event, and 16 myocardial infarction events (95% CI, 6–27) will be prevented. Conversely, nine major bleeding episodes (95% CI, 3–16) and 35 minor bleeding episodes (95% CI, 15–55) would be caused by the antiplatelet therapy. One hundred and sixteen dialysis vascular access failure events (95% CI, 61–180) will also be avoided for every 1000 participants with dialysis vascular access treated for a median of 6 months.

**Table 1:** Events Prevented and Caused by Antiplatelet Therapy for Every 1000 Patients Treated

Outcome	OR (95% CI)	NNT (95% CI)	ARR (95% CI)^a^	CER (%)
Major cardiovascular events	0.85 (0.74,0.94)	44 (26,112)	23 (9,39)	18
Access failure	0.52 (0.31,0.73)	9 (6,16)	116 (61,180)	30
Any bleeding	1.55 (1.25,1.84)	−29 (−62, − 19)	−35 (− 52,-16)	3
Major bleeding	1.33 (1.11,1.59)	− 114 (− 339, − 64)	−9 (− 16, − 3)	6
Minor bleeding	1.66 (1.27,2.05)	−28 (−18, − 68)	−35 (− 55, − 15)	7

*ARR* Absolute risk reduce, *CER* Control event risk, *CI* Confidence interval, *eGFR* estimated glomerular filtration rate, *OR* Odds radio, *NNT* Number needed to be treated, i.e. the number of patients who must be treated to prevent one adverse event

^a^Values are absolute risk change (95% CI) of outcome per 1,000 patients treated for a median follow-up duration. Positive values represent the benefits from antiplatelet therapy

### Sensitivity analysis

Results of all outcomes were robust and none of the sensitivity analyses led to any important changes (Additional file 15: Table S4).

### Evidence quality and publication bias

Summary of evidence quality for all outcomes is presented in Additional file 16: Table S5. The GRADE level showed that the quality of evidence was low in outcome of cardiovascular events, all-cause death, access failure, major and minor bleeding, and very low in kidney failure events, serum creatinine, eGFR and proteinuria. Egger’s test and visual inspection of funnel plots did not suggest publication bias (Additional file 17: Figure S9)

## Discussion

The benefits of antiplatelet therapy for patients with CKD have been debated intensively over recent years. Our systematic review and meta-analysis showed that antiplatelet therapy produces a significant 15% reduction in major cardiovascular events and a mild reduction in proteinuria (0.90 units of SD), compared with placebo or usual-care control groups. For CKD patients requiring or near hemodialysis, antiplatelet agents nearly halve the odds of vascular access failure. These beneficial effects were achieved at the cost of a significant increase in bleeding complications, including major and minor bleeding. However, the net absolute effect suggested that major events, such as cardiovascular events and access failure with antiplatelet agents, outweigh the risk of bleeding. There seemed to be an overall net benefit. No significant effect was observed on the risk of all-cause mortality, kidney failure events, change of serum creatinine, and eGFR. These results were consistent across pre-specified major patient subgroups, types of interventions, and follow-up time.

Comparted with the previous review published in 2012 [30], the current review included many new trials [70, 85, 91] and increased by 44% of the numbers of events available. Major cardiovascular events, including myocardial infarction, stroke, coronary artery revascularization, or cardiovascular death, were used to maximize the power. As a subset of the combined outcome, myocardial infarction was explored as a significant risk reduction from antiplatelet agents (OR: 0.77), while the other components of the primary end point, including stroke, coronary revascularization, and cardiovascular death, tended to decrease; however, they did not reach statistical significance. In terms of all-cause death, although antiplatelet therapy was associated with a non-significant 13% proportional reduction, these results did not provide reliable evidence of a lack of worthwhile benefit in patients with CKD. Insufficient statistical power associated with the low number of events might cause this study not to detect a modest proportional risk reduction of these outcomes. Consistent with our results, the HOT study involving almost 3700 participants with CKD (no-limited kidney function) and hypertension demonstrated the benefits of aspirin, and an increased risk of major bleeding appears to be outweighed by the substantial benefits as well (32). Conversely, the CREDO trial concluded that clopidogrel therapy did not significantly reduce the risk of death, myocardial infarction, or stroke along with increased relative risk of major or minor bleeding compared with placebo. It should be noted that the CKD patients with creatinine above 3 mg/dL were excluded from the CREDO trial [10].

Antiplatelet agents have another role in patients with CKD to prevent dialysis vascular access thrombosis by the blockade of platelet activation and aggregation in some trials [47, 78]. Our study suggested that antiplatelet therapy reduced the odds of vascular access failure events by 48%, which is similar to the previous meta-analysis [9]. However, moderate heterogeneity (*I*^*2*^ = 46%) was found. The different types of drugs seemed to explain the part source of heterogeneity in subgroup analysis. A key issue remains that antiplatelet therapy has uncertain side-effects with unclear effects on major bleeding in CKD stage 5, although the previous meta-analysis [9] and our subgroup results of CKD stage 5 did not find a significantly increased risk of bleeding. What must be noted is that the low numbers of events for bleeding outcomes in patients with CKD stage 5 resulted in imprecise risk effect estimates, that is, for these population summary risks of bleeding provided by randomized trials currently could be unreliable and uncertain. The well-conducted trial by Kaufman et al. [72] was terminated early because of increased risk of bleeding in those patients with CKD stage 5 who received dual-antiplatelet therapy. These results suggested that the risk of bleeding should be considered cautiously by clinicians before prescription.

Although this study did not show a clear renal benefit from antiplatelet therapy in patients with CKD, it is important that there was a lack of evidence for an adverse effect of antiplatelet agents on kidney outcomes, particularly in light of the potential benefits of proteinuria. Consistent with our study, the UK-HARP-I [73] and HOT study [33] showed that using aspirin in patients with CKD was not associated with progression of CKD. However, only a few trails reported kidney outcomes. We found significant heterogeneity for the continuous outcomes in the current study, based on six trials and 120 kidney failure events. The results in terms of kidney outcomes from antiplatelet therapy should be interpreted with caution.

Furthermore, there are many open questions about antiplatelet therapy in populations with CKD, which might help to establish the basis for future research. First, because of the missing baseline eGFR value in most original studies, we could only divide the patients into the subgroups of CKD stage 5 and not stage 5. No statistical difference was observed for the main outcomes between CKD stage 5 and no-5 stage. However, the HOT study demonstrated greater proportional benefits with progressively lower eGFR [33]. It is still uncertain whether the effect of antiplatelet therapy is modified by kidney function similar to statin in CKD patients [94] or whether the complicated non-linear relations between eGFR and cardiovascular events exist [95]. Further trials should try to address the outstanding issue. Second, although this review concluded that there was a net benefit of preventing 23 major cardiovascular events in 1000 patients using antiplatelet agents versus incurring nine major bleeding events, it would seem unacceptable for most patients and clinicians in fatal bleeding, such as severe intracranial hemorrhage occurred. Additionally, the significant risk reduction in all-cause deaths was not still proven. Thus, among patients with CKD, determining the trade-off between the benefits and risks of antiplatelet therapy remains challenging. An individualized assessment and balancing of bleeding and ischemic risks should be mandatory. An integral evaluation and monitoring system are needed to guide clinical practice. Third, due to the lack of head-to-head comparisons between antiplatelet agents, the current study cannot exclude the possibility of a more moderate protective effect by some antiplatelet agents in patients with CKD. This implies heterogeneity in terms of the effects of cardiovascular protection and risk of bleeding among different types of antiplatelet drugs. The PLATO trial also suggested that ticagrelor compared with clopidogrel reduced major cardiovascular events and all-cause death, with no significant increase in major or fatal bleedings in acute coronary syndrome patients with creatinine clearance less than 60 mL/min [13]. Future studies with head-to-head comparisons of antiplatelets drugs are needed to illuminate whether important differences exist in their cardiovascular protection and bleeding risk abilities, and whether dose, types or combination regimen matter.

The study does have some potential limitations. First, unpublished data about CKD patients in several large trials derived from previous meta-analysis, although they were reported after confirmation. Most large RCTs of antiplatelet therapy were not primarily designed to assess outcomes in subjects presenting with CKD. These post-hoc analyses and unpublished data maybe limited the reliability of the conclusions drawn. Second, the existences of statistical heterogeneity and the inevitable clinical heterogeneity might raise critical concerns regarding validity. Heterogeneity in the study population as included in the umbrella term ‘CKD patients’ remains a big concern for meaningful interpretation of the results. Therefore, our study should be considered hypothesis generating and requires further research. Third, we used trial-level data because patient-level data were not available, which would have allowed a more reliable assessment of treatment effects in different patient groups. Fourth, the quality of evidence was weak. Our results provide hypothesis-generating, rather than confirmatory, evidence for antiplatelet treatment effects and adverse events in population with CKD. Further studies are warranted to confirm them.

## Conclusions

This review suggested that antiplatelet therapy might reduce the occurrence of major cardiovascular events and hemodialysis vascular access failure in CKD patients compared with placebo or usual-care groups. However, the significantly increased risk of bleeding should be considered. Although there is seemingly a net benefit of using antiplatelet therapy for CKD patients, complete evaluation and careful monitoring should permeate the whole therapy process. Physicians should weigh the tradeoff between benefits and risks of bleeding individually. Further antiplatelet studies are warranted to confirm and integrate these results for CKD patients.

## Additional files

Additional file 1: Search Strategy. (DOCX 24 kb)

Additional file 2: Assessment domains of risk of bias. (DOCX 16 kb)

Additional file 3: The codes of Winbugs for the full Bayes methods. (DOCX 14 kb)

Additional file 4: **Table S1.** Summary of Characteristics of Included Trials and Patients. (DOCX 15 kb)

Additional file 5: **Table S2.** Characteristics of Included Trials and Patients. (DOCX 33 kb)

Additional file 6: **Figure S1.** Risk of bias graph. (DOCX 55 kb)

Additional file 7: **Figure S2.** Risk of bias summary. (DOCX 975 kb)

Additional file 8: **Figure S3.** Forest plot for all-cause death and cardiovascular death. (DOCX 102 kb)

Additional file 9: **Table S3.** Subgroup Analysis of Outcomes and Adverse Events. (DOCX 20 kb)

Additional file 10: **Figure S4.** Forest plot for the rate of eGFR decline. (DOCX 54 kb)

Additional file 11: **Figure S5.** Forest plot for the change of serum creatinine. (DOCX 44 kb)

Additional file 12: **Figure S6.** Forest plot for the change of proteinuria or albuminuria. (DOCX 52 kb)

Additional file 13: **Figure S7.** Forest plot for hemodialysis vascular access. (DOCX 67 kb)

Additional file 14: **Figure S8.** Forest plot for adverse events (major and minor Bleeding). (DOCX 61 kb)

Additional file 15: **Table S4.** Sensitivity Analysis of Outcomes and Adverse Events. (DOCX 20 kb)

Additional file 16: **Table S5.** Evidence Profile for the Effect of Antiplatelet Therapy on Outcomes in Patients With CKD. (DOCX 17 kb)

Additional file 17: **Figure S9.** Funnel plots and Egger’s test for small study effects. (DOCX 29 kb)

## Data Availability

All data generated or analyzed during this study are included in this article and its additional files.

## Abbreviations

CI: Confidence intervals
CKD: Chronic kidney disease
CVD: Cardiovascular disease
eGFR: estimated glomerular filtration rate
ESRD: End-stage renal disease
NNT: Number needed to be treated
OR: Odds ratio
RCT: Random control trial
