# Supplementary material for: Effect of antiplatelet therapy on cardiovascular and kidney outcomes in patients with chronic kidney disease: a systematic review and meta-analysis
Source: BMC Nephrol. 2019 Aug 7;20:309. doi: 10.1186/s12882-019-1499-3 (PMC6686545; doi:10.1186/s12882-019-1499-3)
Supplement: Supplementary file 1 — Search Strategy. (DOCX 24 kb) [file 12882_2019_1499_MOESM1_ESM.docx]

**Additional file 1*.*** **Search Strategy**

**1)** **Ovid MEDLINE** (**1946 to Dec 2018)**

1. exp Platelet Aggregation Inhibitors/
2. platelet glycoprotein adj5 blockade.mp.
3. platelet glycoprotein adj5 inhibit$.mp.
4. platelet aggregation inhibit$.mp.
5. thrombocyte aggregation inhibit$.mp.
6. (adenosine diphosphate receptor inhibit$ or ADP receptor inhibit$).mp.
7. (adenosine reuptake inhibit$ or adenosine re-uptake inhibit$).mp.
8. antiplatelet or anti-platelet.mp.
9. (P2Y12 adj2 antagonis$).mp.
10. exp Aspirin/
11. aspirin$.mp.
12. acetylsalicylic acid$.mp.
13. acid, acetylsalicylic.mp.
14. 2-acetyloxy benzoic acid$.mp.
15. (acylpyrin$ or aloxiprimum$ or colfarit$ or dispril$ or easprin$ or ecotrin$ or endosprin$ or magnecyl$ or micristin$ or polopirin$ or polopiryna$ or solprin$ or solupsan$ or zorprin$ or acetysal$).mp.
16. (dipyridamole or ticlopidine or clopidogrel or sulfinpyrazone or sulphinpyrazone or cilostazol or prasugrel or ticagrelor or cangrelor or elinogrel or abciximab or eptifibatide or tirofiban or defibrotide or picotamide or beraprost or ticlid or aggrenox or ditazole or vorapaxar or atopaxar).mp.
17. or/1-16
18. exp Kidney Disease/
19. kidney or renal.mp.
20. nephropathy.mp.
21. exp Renal Insufficiency, Chronic/
22. chronic kidney disease.mp.
23. exp Renal Dialysis/
24. exp Renal Replacement Therapy/
25. (hemodialysis or haemodialysis).mp.
26. (hemofiltration or haemofiltration).mp.
27. (hemodiafiltration or haemodiafiltration).mp.
28. dialysis.mp.
29. (PD or CAPD or CCPD or APD or IPD).mp.
30. (CKF or CKD or CRF or CRD).mp.
31. (end-stage renal or end-stage kidney or end stage renal or end stage kidney).mp.
32. (ESRF or ESKF or ESRD or ESKD).mp.
33. exp Kidney Transplantation/
34. Uremia/
35. ur?emi$.mp.
36. or/18-35
37. randomized controlled trial.pt.
38. controlled clinical trial.pt.
39. randomized.ab.
40. placebo.ab.
41. clinical trials as topic.sh.
42. randomly.ab.
43. trial.ti.
44. or/21-27
45. (animals not (humans and animals)).sh.
46. 44 not 45
47. 17 and 36 and 46

**2) Embase (1966-Dec 2018)**

1. 'platelet aggregation inhibitors'/exp OR 'platelet aggregation inhibitors'
2. 'platelet glycoprotein' NEAR/5 blockade
3. 'platelet glycoprotein' NEAR/5 inhibit*
4. 'thrombocyte aggregation inhibit*'
5. 'adenosine diphosphate receptor inhibit*' OR 'adp receptor inhibit*'
6. 'adenosine reuptake inhibit*' OR 'adenosine re-uptake inhibit*'
7. antiplatelet OR 'anti platelet'
8. p2y12 NEAR/2 antagonis*
9. 'aspirin'/exp OR aspirin
10. 'acetylsalicylic acid*'
11. '2-acetyloxy benzoic acid*'
12. acylpyrin* OR aloxiprimum* OR colfarit* OR dispril* OR easprin* OR ecotrin* OR endosprin* OR magnecyl* OR micristin* OR polopirin* OR polopiryna* OR solprin* OR solupsan* OR zorprin* OR acetysal*
13. dipyridamole* OR ticlopidine* OR clopidogrel* OR sulfinpyrazone* OR sulphinpyrazone* OR cilostazol* OR prasugrel* OR ticagrelor* OR cangrelor* OR elinogrel* OR abciximab* OR eptifibatide* OR tirofiban* OR defibrotide* OR picotamide* OR beraprost* OR ticlid* OR aggrenox* OR ditazole* OR vorapaxar* OR atopaxar*
14. #1 OR #2 OR #3 OR #4 OR #5 OR #6 OR #7 OR #8 OR #9 OR #10 OR #11 OR #12 OR #13
15. 'kidney disease'/exp OR 'kidney disease'
16. kidney OR renal
17. nephropathy
18. 'renal insufficiency, chronic'/exp OR 'renal insufficiency, chronic'
19. 'chronic kidney disease'
20. 'renal dialysis'/exp OR 'renal dialysis'
21. 'renal replacement therapy'/exp OR 'renal replacement therapy'
22. hemodialysis OR haemodialysis
23. hemofiltration OR haemofiltration
24. hemodiafiltration OR haemodiafiltration
25. dialysis
26. pd OR capd OR ccpd OR apd OR ipd
27. ckf OR ckd OR crf OR crd
28. 'end-stage renal' OR 'end-stage kidney' OR 'end stage renal' OR 'end stage kidney'
29. esrf OR eskf OR esrd OR eskd
30. 'kidney transplantation'/exp OR 'kidney transplantation'
31. 'uremia'/exp OR uremia
32. ur?emi*
33. #15 OR #16 OR #17 OR #18 OR #19 OR #20 OR #21 OR #22 OR #23 OR #24 OR #25 OR #26 OR #27 OR #28 OR #29 OR #30 OR #31 OR #32
34. random* OR blind* OR placebo OR 'meta analysis'
35. #14 AND #33 AND #34

**3) CENTRAL (on Dec, 2018)**

#1 Platelet Aggregation Inhibitors

#2 MeSH descriptor: [Platelet Aggregation Inhibitors] explode all trees

#3 'platelet glycoprotein' near/5 blockade

#4 'platelet glycoprotein' near/5 inhibit*

#5 thrombocyte aggregation inhibit*

#6 'adenosine diphosphate receptor inhibit*' or 'adp receptor inhibit*'

#7 'adenosine reuptake inhibit*' or 'adenosine re-uptake inhibit*'

#8 antiplatelet or 'anti platelet'

#9 p2y12 near/2 antagonis*

#10 MeSH descriptor: [Aspirin] explode all trees

#11 aspirin*

#12 acetylsalicylic acid*

#13 2-acetyloxy benzoic acid*

#14 acylpyrin* or aloxiprimum* or colfarit* or dispril* or easprin* or ecotrin* or endosprin* or magnecyl* or micristin* or polopirin* or polopiryna* or solprin* or solupsan* or zorprin* or acetysal*

#15 dipyridamole* or ticlopidine* or clopidogrel* or sulfinpyrazone* or sulphinpyrazone* or cilostazol* or prasugrel* or ticagrelor* or cangrelor* or elinogrel* or abciximab* or eptifibatide* or tirofiban* or defibrotide* or picotamide* or beraprost* or ticlid* or aggrenox* or ditazole* or vorapaxar* or atopaxar*

#16 #1 or #2 or #3 or #4 or #5 or #6 or #7 or #8 or #9 or #10 or #11 or #12 or #13 or #14 or #15

#17 MeSH descriptor: [Kidney Diseases] explode all trees

#18 kidney disease*

#19 kidney or renal

#20 nephropathy

#21 MeSH descriptor: [Renal Insufficiency, Chronic] explode all trees

#22 renal insufficiency, chronic

#23 chronic kidney disease

#24 renal dialysis

#25 MeSH descriptor: [Renal Dialysis] explode all trees

#26 MeSH descriptor: [Renal Replacement Therapy] explode all trees

#27 renal replacement therap*

#28 hemodialysis or haemodialysis

#29 hemofiltration or haemofiltration

#30 hemodiafiltration or haemodiafiltration

#31 dialysis

#32 pd or capd or ccpd or apd or ipd

#33 ckf or ckd or crf or crd

#34 'end-stage renal' or 'end-stage kidney' or 'end stage renal' or 'end stage kidney'

#35 esrf or eskf or esrd or eskd

#36 MeSH descriptor: [Kidney Transplantation] explode all trees

#37 kidney transplantation

#38 MeSH descriptor: [Uremia] explode all trees

#39 ur?emi*

#40 #17 or #18 or #19 or #20 or #21 or #22 or #23 or #24 or #25 or #26 or #27 or #28 or #29 or #30 or #31 or #32 or #33 or #34 or #35 or #36 or #37 or #38 or #39

#41 #16 and #40
