# Supplementary material for: Effect of antiplatelet therapy on cardiovascular and kidney outcomes in patients with chronic kidney disease: a systematic review and meta-analysis
Source: BMC Nephrol. 2019 Aug 7;20:309. doi: 10.1186/s12882-019-1499-3 (PMC6686545; doi:10.1186/s12882-019-1499-3)
Supplement: Supplementary file 3 — The codes of Winbugs for the full Bayes methods. (DOCX 14 kb) [file 12882_2019_1499_MOESM3_ESM.docx]

**Additional file 3: The codes of Winbugs for the full Bayes method.**

model

{

for (i in 1:k)

{

rc[i] ~ dbin(pic[i],nc[i])

rt[i] ~ dbin(pit[i],nt[i])

mu[i] <- logit(pic[i])

logit(pit[i]) <- mu[i] + delta[i]

delta[i] ~ dnorm(delt,precision.tau)

pic[i] ~ dunif(0,1)

}

delt ~ dnorm(0,0.00001)

precision.tau <- 1/tau.squared

tau.squared <- tau*tau

tau ~ dunif(0,2)

OR<-exp(delt)

}
