# Supplementary material for: Effect of antiplatelet therapy on cardiovascular and kidney outcomes in patients with chronic kidney disease: a systematic review and meta-analysis
Source: BMC Nephrol. 2019 Aug 7;20:309. doi: 10.1186/s12882-019-1499-3 (PMC6686545; doi:10.1186/s12882-019-1499-3)
Supplement: Supplementary file 4 — Table S1. Summary of Characteristics of Included Trials and Patients. (DOCX 15 kb) [file 12882_2019_1499_MOESM4_ESM.docx]

**Additional file 4:** **Table S1.** Summary of Characteristics of Included Trials and Patients

| **Outcomes** | **No. of Studies/ No. of Patients** | **Sample Size** | **Follow-up, month** | **Age, y** | **Male Sex** |
| --- | --- | --- | --- | --- | --- |
| **Major cardiovascular events** | 25/25315 | 504(16-4849) | 12(1-95) | 61(51-75) | 59.5% |
| **All-cause death** | 24/24708 | 468 (16-4849) | 12 (3-60) | 59 (51-70) | 57.1% |
| **Access failure** | 15/2998 | 93 (16-866) | 6 (1-60) | 51 (44-58) | 51.1% |
| **Kidney failure events** | 7/811 | 59 (27-448) | 18 (12-60) | 63 (31-71) | 63.8% |
| **Any Bleeding** | 29/26118 | 448(36-4849) | 12(1-60) | 61(31-75) | 55.0% |
| **Major Bleeding** | 27/25815 | 488 (36-4849) | 12 (1-60) | 65 (44-75) | 54.8% |
| **Minor Bleeding** | 23/23138 | 468 (36-4849) | 12 (1.5-60) | 61 (31-75) | 54.6% |
| **Serum creatinine** | 4/144 | 25 (20-76) | 18 (2-36) | 51 (31-57) | 73.8% |
| **eGFR** | 7/3934 | 62 (22-3619) | 12 (2-54) | 57 (37-66) | 53.0% |
| **Proteinuria** | 8/367 | 36 (20-90) | 12 (2-54) | 51 (31-66) | 61.7% |

Unless otherwise indicated, values are given as median (range).

eGFR = estimated glomerular filtration rate; ESRD = end stage renal disease
