# Supplementary material for: Effect of antiplatelet therapy on cardiovascular and kidney outcomes in patients with chronic kidney disease: a systematic review and meta-analysis
Source: BMC Nephrol. 2019 Aug 7;20:309. doi: 10.1186/s12882-019-1499-3 (PMC6686545; doi:10.1186/s12882-019-1499-3)
Supplement: Supplementary file 5 — Table S2. Characteristics of Included Trials and Patients. (DOCX 33 kb) [file 12882_2019_1499_MOESM5_ESM.docx]

| **Additional file 5: Table S2*.*** Characteristics of Included Trials and Patients | | | | | | | | | | | | | |  |
| --- | --- | --- | --- | --- | --- | --- | --- | --- | --- | --- | --- | --- | --- | --- |
| **Study** | **Inclusion criteria** | **Outcome** | **Number of patients** | **Mean follow-up**  **months** | **Men**  **(%)** | **Mean age, y** | **Definition of CKD** | **Treatment group** | **Control group** | **Funding Source** | **Data source** | **Nonrandomized Co-Interventions** | **Jadad score** | |
| **Anderson 1974** | kidney transplant patients | graft loss; transplant rejection | 27 | 24 | NA | NA | renal transplantation | dipyridamole | placebo | NA | full text | - | 1 | |
| **Andrassy 1974** | patients with ESRD | access failure; major and minor bleeding | 92 | 1 | NA | NA | ESRD | aspirin | placebo | NA | full text | - | 2 | |
| **Kaegi 1974** | patients with straight AV shunts | access failure; major bleeding | 62 | 6 | 69 | 44 | ESRD | sulfinpyrazone | placebo | Non-industry with drug supplied by industry | full text | - | 4 | |
| **Michie 1977** | scheduled to begin haemodialysis | all-cause death; cardiovascular death; access failure | 16 | 3 | 63 | 51 | ESRD | sulfinpyrazone | placebo | NA | full text | - | 3 | |
| **Donadio 1984** | biopsy proven MPGN | kidney failure events; minor bleeding; | 50 | 12 | 58 | 31 | non-dialysis | dipyridamole+aspirin | placebo | Non-industry with drug supplied by industry | full text | - | 4 | |
| **Harter 1979** | patients receiving AV shunts | access failure | 44 | 4.7 | 47 | 50 | ESRD | aspirin | placebo | Non-industry with drug supplied by industry | full text | - | 3 | |
| **Kobayashi 1980** | haemodialysis patients with AV external shunts or vascular grafts who had experienced more than one episode of thrombosis of their fistula during the preceding 4 weeks | major and minor bleeding | 107 | 3 | 37 | NA | ESRD | ticlopidine | placebo | NA | full text | - | 3 | |
| **ELL 1982** | patients with haemodialysis | all-cause death; cardiovascular death | 50 | 3 | NA | NA | ESRD | ticlopidine | usual treatment | NA | meta-analysis | - | 1 | |
| **Nyberg 1984** | DN | change of eGFR | 22 | 12 | NA | NA | DN | ticlopidine | placebo | NA | full text | - | 3 | |
| **Fiskerstrand 1985** | patients requiring access surgery for haemodialysis | access failure | 18 | 1 | NA | NA | ESRD | ticlopidine | placebo | NA | full text | - | 2 | |
| **Grontoft 1985** | patients receiving AVF surgery | access failure; major and minor bleeding | 36 | 1 | 67 | 45 | ESRD | ticlopidine | placebo | NA | full text | - | 3 | |
| **Creek 1990** | patients with haemodialysis | fatal/nonfatal MI; all-cause death; major bleeding; cardiovascular death | 285 | 5 | NA | NA | ESRD | ticlopidine | usual treatment | NA | meta-analysis | - | 1 | |
| **Schulze 1990** | patients with kidney transplant | graft loss; kidney failure events | 64 | 12 | NA | NA | renal transplantation | dipyridamole | no-treatment | NA | full text | - | 1 | |
| **ETDRS 1992** | 18 to 70 years of age; DM and diabetic retinopathy; Scr> 133μmol/L | fatal/nonfatal MI; stroke; all-cause death; cardiovascular death; kidney failure events | 185 | 60 | 71 | 54 | non-dialysis | aspirin | placebo | non-industry | meta-analysis | - | 4 | |
| **Middleton 1992** | patients with haemodialysis | all-cause death; cardiovascular death; major bleeding | 903 | 18 | NA | NA | ESRD | asprin and dipyridamole | usual treatment | NA | meta-analysis | - | 1 | |
| **Kooistra 1994** | aged ≥ 18 years; chronic dialysis for more than 6 weeks | access failure; minor bleediing | 137 | 6 | NA | NA | ESRD | aspirin | placebo | NA | full text | - | 2 | |
| **Sreedhara 1994** | haemodialysis patients with PTFE graft | all-cause death; access failure; major bleeding | 107 | 18 | 36 | 55 | ESRD | aspirin and/or dipyridamole | placebo | industry | full text | - | 4 | |
| **EPIC 1994** | patients scheduled to undergo coronary angioplasty or directional atherectomy with high risk for abrupt vessel closure | fatal/nonfatal MI; stroke; all-cause death; major and minor bleeding; coronary revasularization | 519 | 12 | NA | 61 | eGFR<60ml/min/1.73m^2^ | abciximab (c7E3b) | placebo | non-industry | meta-analysis | aspirin plus heparin | 5 | |
| **Zauner 1994** | biopsy proven MPGN and nephrotic syndrome | change of Scr and proteinuria | 18 | 36 | 61 | 45 | MPGN | aspirin and dipyridamole | placebo | NA | full text | - | 2 | |
| **STOP 1995** | haemodialysis patients (at least 60 days) with permanent internal stabilised vascular access | all-cause death; cardiovascular death; stroke; major bleeding | 811 | 12 | NA | NA | ESRD | picotamide | placebo | NA | meta-analysis | - | 4 | |
| **Frascra 1996** | patients with IgAN and SCr ≥1.4mg/dL | change of Scr and proteinuria | 20 | 24 | 90 | 31 | IgAN | defibrotide | usual treatment | NA | full text | prednisone | 1 | |
| **EPILOG 1997** | age≥21 years; patients undergoing elective or urgent PCI | fatal/nonfatal MI; stroke; all-cause death; major and minor bleeding; coronary revasularization | 488 | 12 | NA | 60 | eGFR<60ml/min/1.73m^2^ | abciximab (c7E3b) | placebo | industry | meta-analysis | aspirin plus heparin | 5 | |
| **IMPACT-II 1997** | patients were scheduled for elective, urgent, or emergency PCI | fatal/nonfatal MI; all-cause death; major and minor bleeding; coronary revasularization | 806 | 6 | NA | 61 | eGFR<60ml/min/1.73m^2^ | eptifibatide | placebo | industry | meta-analysis | aspirin plus heparin | 5 | |
| **RAPPORT 1998** | patients within 12 hours of the onset of AMI, referred for primary angioplasty | fatal/nonfatal MI; stroke; all-cause death; major and minor bleeding; coronary revasularization | 62 | 6 | 73 | 61 | eGFR<60ml/min/1.73m^2^ | abciximab (c7E3b) | placebo | industry | meta-analysis | aspirin | 4 | |
| **Cheng 1998** | 21 to 65 years of age; biopsy proven IgAN with at least 2 suggestive of progressive disease | kidney failure events; change of proteinuria and eGFR | 31 | 54 | 55 | 37 | IgAN | ticlopidine | usual treatment | non-industry | full text | captopril | 2 | |
| **EPISTENT 1998** | patients undergoing elective or urgent PCI | fatal/nonfatal MI; stroke; all-cause death; major and minor bleeding; coronary revasularization | 368 | 12 | NA | 59 | eGFR<60ml/min/1.73m^2^ | abciximab (c7E3b) | placebo | Non-industry with drug supplied by industry | meta-analysis | aspirin plus heparin | 5 | |
| **Giustina 1998** | 40 to 65 years of age; DM and diabetic retinopathy; Scr<106μmol/L; 24hUAE 20-200μg/min; HbA1C<10%; supine BP 140/90mmHg | change of Scr, Crcl and proteinuria | 30 | 12 | 87 | 57 | DN | picotamide | placebo | non-industry | full text | - | 4 | |
| **Grontoft 1998** | patients with chronic renal failure predialysis or when on dialysis who had been selected for surgery for an AVF, saphenous or artificial grafts as hemodialysis access site | access failure; all-cause death; cardiovascular death | 242 | 1 | 63 | 57 | ESRD | ticlopidine | placebo | non-industry | full text | - | 5 | |
| **PURSUIT 1998** | ischemic chest pain at rest lasting 10 min or longer within the previous 12h; change of electrocardiogram of ACS | fatal/nonfatal MI; stroke; all-cause death; major and minor bleeding; coronary revasularization | 2617 | 6 | NA | 64 | eGFR<60ml/min/1.73m^2^ | eptifibatide | placebo | non-industry | full text | aspirin plus heparin | 5 | |
| **UK-HARP-I 2005** | aged ≥ 18 years; predialysis patients with Scr ≥1.7mg/dL or chronic dialysis patients or functioning kidney transplant | fatal/nonfatal MI; stroke; all-cause death; cardiovascular death; major and minor bleeding; coronary revasularization; kidney failure events | 448 | 12 | 71 | 53 | Scr≥150umol/L | aspirin | placebo | industry | full text | simvastatin | 5 | |
| **PRISM-PLUS 2002** | patients with ACS | the composite end points of death/MI/refractory ischemia; major and minor bleeding | 611 | 6 | 45 | 75 | eGFR<60ml/min//1.73m^2^ | tirofiban | placebo | NA | full text | heparin | 4 | |
| **Khajehdehi 2002** | T2DM; normal kidney function; UAE>500mg/d; not receiving ACEI | change of eGFR, Scr and proteinuria | 76 | 2 | 53 | 57 | DN and Scr<2mg/dL | aspirin or/and dipyridamole | placebo | NA | full text | - | 2 | |
| **Kaufman 2003** | haemodialysis patients with PTFE graft in the arm | fatal/nonfatal MI; stroke; all-cause death; major and minor bleeding; access failure | 200 | 12 | 99 | 62 | ESRD | aspirin and clopidogrel | placebo | Non-industry with drug supplied by industry | full text and meta analysis | - | 5 | |
| **Abdul-Rahman 2007** | patients with tunneled central venous catheter | access failure | 38 | 12 | 42 | 45 | ESRD | aspirin | no-treatment | NA | full-text | - | 2 | |
| **CURE 2007** | ACS patients hospitalized within< 24h of symptom and without ST-segment elevation | all-cause death; cardiovascular death; major and minor bleeding; the composite end points of cardiovascular death/MI/stroke | 4087 | 12 | 49 | 70 | eGFR<64ml/min/1.73m^2^ | clopidogrel | placebo | non-industry | full-text | aspirin | 5 | |
| **CREDO 2008** | age≥ 21 years; symptomatic coronary attery disease; elective PCI planned or considered | fatal/nonfatal MI; stroke; all-cause death; cardiovascular death; major and minor bleeding; coronary revasularization | 411 | 12 | NA | 74 | eGFR<60ml/min/1.73m^2^ | clopidogrel | placebo | non-industry | full-text and meta-analysis | aspirin | 5 | |
| **Dember 2008** | patients with haemodialysis or that were expected to begin haemodialysis within 6M | fatal/nonfatal MI; stroke; all-cause death; major and minor bleeding; access failure | 877 | 1.5 | 62 | 53 | ESRD | clopidogrel | placebo | non-industry | full text and meta-analysis | aspirin | 5 | |
| **CHARISMA 2009** | age≥45 years and diabetic kidney disease | fatal/nonfatal MI; stroke; all-cause death; cardiovascular death; major and minor bleeding | 2009 | 28 | 67 | 63 | DN | clopidogrel | placebo | non-industry | full-text | aspirin | 5 | |
| **Dixon 2009** | age≥18 years; patients with haemodialysis or that were scheduled to begin haemodialysis within 6M | fatal/nonfatal MI; stroke; all-cause death; major and minor bleeding; access failure | 649 | 60 | 40 | 58 | ESRD | dipyridamole and aspirin | placebo | non-industry | full-text | - | 5 | |
| **Ghorbani 2009** | age>18 years; patients close to hemodialysis requiring AVF, and haemodialysis patients requiring a new AVF | all-cause death; major bleeding; access failure | 93 | 6 | 26 | 45 | ESRD | clopidogrel | placebo | non-industry | full-text | - | 4 | |
| **HOT 2010** | 40 to 65 years of age; DBP between 100 and 115mmHg | fatal/nonfatal MI; stroke; all-cause death; cardiovascular death; major and minor bleeding | 3619 | 45.6 | 34 | 65 | eGFR<60ml/min//1.73m^2^ | aspirin | placebo | non-industry | full-text | - | 5 | |
| **Luk 2010** | T2DM with ACR ≥ 30 mg/mmol | change of eGFR and proteinuria | 62 | 12 | NA | NA | DN | cilostazol | placebo | NA | abstract | - | 4 | |
| **Rouzrokh 2010** | patients who needed AVF for heamodialysis | access failure | 390 | 6 | 47 | NA | ESRD | dipyridamole or aspirin | placebo | non-industry | full-text | - | 3 | |
| **JPAD 2011** | 30 to 85 years of age; T2DM patients without any history of atherosclerotic disease | major bleeding | 632 | 52.4 | 53 | 68 | eGFR<60ml/min//1.73m^2^ | aspirin | usual treatment | industry | full-text | - | 4 | |
| **Watanable 2011** | diabetic patients with stable angina and CKD | the composite end points of major adverse cardiovascular events or hospitalization for revascularization | 79 | 90 | NA | NA | CKD stage 3 or 4 | sarpogrelate | usual treatment | NA | abstract | - | 2 | |
| **Jiao 2013** | DM with UAE 30-300mg/24h | change of proteinuria | 40 | 6 | 50 | NA | diabetes nephrology | cilostazol | placebo | NA | full-text | - | 3 | |
| **Mozafar 2013** | hemodialysis patients for whom AV access may be delayed until arteriovenous access maturation for dialysis | major bleeding | 180 | NA | 60 | 61 | ESRD | aspirin | placebo | no-industry | full-text | - | 4 | |
| **Tang 2014** | 30 to 85 years of age; DN | change of Scr, eGFR and proteinuria | 90 | 12 | 36 | 66 | diabetes nephrology | cilostazol | placebo | no-industry | full-text | - | 4 | |
| **PEGASUS-TIMI 54 2015** | patients with a spontaneous MI occurring 1–3 years prior to enrolment and Crcl< 60 mL/min | fatal/nonfatal MI; all-cause death; major and minor bleeding; the composite end points of cardiovascular death/MI/stroke | 4849 | 36 | 37 | 70 | eGFR<60ml/min//1.73m^2^ | ticagrelor | placebo | industry | full-text | aspirin | 5 | |
| **AASER Study** | patients without previous cardiovascular events with stage 3 or 4 CKD | cardiovascular death, acute coronary syndrome, heart failure; renal outcome; bleeding | 116 | 65 | 31 | 67 | eGFR 15-60ml/min//1.73m^2^ | aspirin | Standard treatment | none | Full-text | - | 3 | |

ACEI = angiotensin converting enzyme inhibitors; ACS = cute coronary syndrome; ACR = albumin creatinine ratio; AV = arteriovenous; AVF = arteriovenous fistula; CKD = chronic kidney disease; CrCl = creatinine clearance; DBP = Diastolic blood pressure; DM = diabetes mellitus; DN = diabetes nephropathy; eGFR = estimated glomerular filtration rate; ESRD = end stage renal disease; IgAN = IgA nephropathy; PTFE = polytetrafluoroethylene; MI = myocardial infarction; MPGN = Mesangial proliferative glomerulonephritis; NA = no available; Scr = serum creatinine; T2DM = type 2 diabetes mellitus; UAE = urinary albumin excretion.
