# Supplementary figures and images for: Effect of antiplatelet therapy on cardiovascular and kidney outcomes in patients with chronic kidney disease: a systematic review and meta-analysis
Source: BMC Nephrol. 2019 Aug 7;20:309. doi: 10.1186/s12882-019-1499-3 (PMC6686545; doi:10.1186/s12882-019-1499-3)

**Additional file 6: Figure S1.** Risk of bias graph


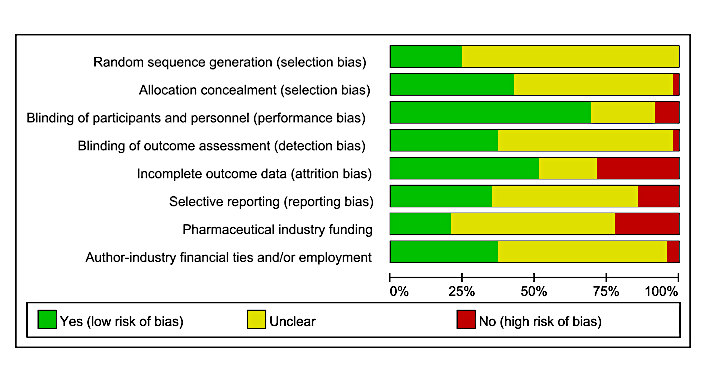

Supplement: Supplementary file 6 — Figure S1. Risk of bias graph. (DOCX 55 kb) [file 12882_2019_1499_MOESM6_ESM.docx]

**Additional file 7: Figure S2.** Risk of bias summary


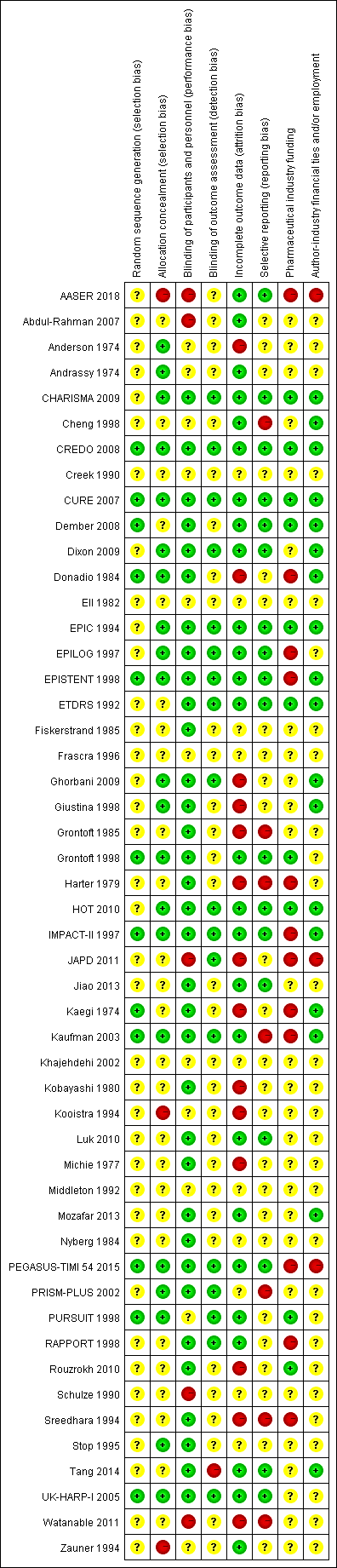

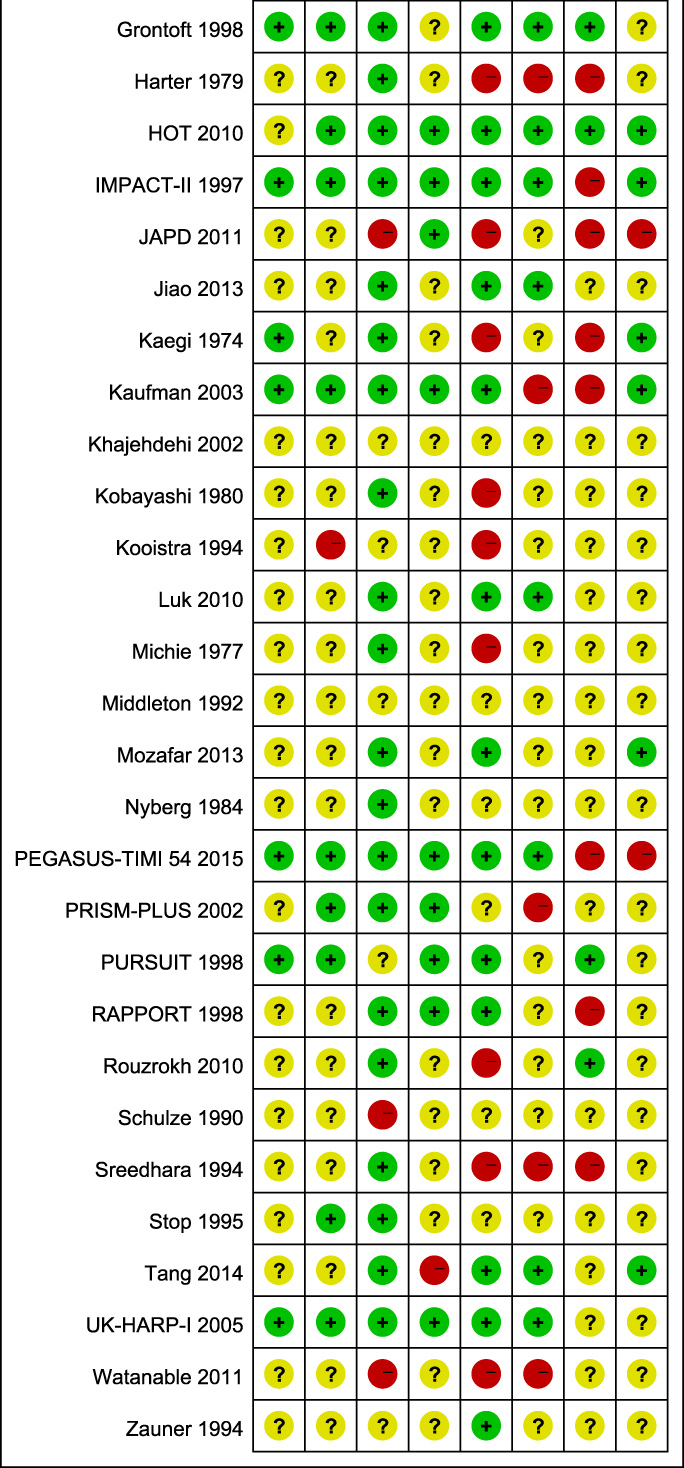

Supplement: Supplementary file 7 — Figure S2. Risk of bias summary. (DOCX 975 kb) [file 12882_2019_1499_MOESM7_ESM.docx]
