# Supplementary material for: Effect of antiplatelet therapy on cardiovascular and kidney outcomes in patients with chronic kidney disease: a systematic review and meta-analysis
Source: BMC Nephrol. 2019 Aug 7;20:309. doi: 10.1186/s12882-019-1499-3 (PMC6686545; doi:10.1186/s12882-019-1499-3)
Supplement: Supplementary file 8 — Figure S3. Forest plot for all-cause death and cardiovascular death. (DOCX 102 kb) [file 12882_2019_1499_MOESM8_ESM.docx]

**Additional file 9: Figure S3.** Forest plot for all-cause death and cardiovascular death**.**

HOT 2010

Dember 2008

Ghorbani 2009

PEGASUS-TIMI 54 2015

ETDRS 1992

CHARISMA 2009

Dixon 2009

CREDO 2008

Sreedhara 1994

EPILOG 1997

PURSUIT 1998

Grontoft 1998

EPISTENT 1998

EPIC 1994

STOP 1995

RAPPORT 1998

Kaufman 2003

Michie 1977

IMPACT-II 1997

0.74 (0.53, 1.04)

0.99 (0.25, 3.98)

1.02 (0.14, 7.58)

0.91 (0.73, 1.12)

1.20 (0.67, 2.16)

1.67 (1.14, 2.44)

0.90 (0.65, 1.25)

1.30 (0.50, 3.35)

0.35 (0.07, 1.71)

0.60 (0.24, 1.48)

1.05 (0.82, 1.34)

0.50 (0.09, 2.78)

0.84 (0.31, 2.26)

1.07 (0.52, 2.20)

0.93 (0.47, 1.81)

0.39 (0.07, 2.09)

0.68 (0.15, 3.13)

0.29 (0.01, 8.37)

0.49 (0.23, 1.04)

62/1791

4/441

2/46

266/3200

46/79

73/1006

105/321

10/203

4/83

11/325

161/1430

2/129

10/231

23/334

17/398

2/27

3/104

0/8

15/547

84/1828

4/436

2/47

150/1649

57/106

45/1003

115/328

8/208

3/24

9/163

127/1176

4/131

7/137

12/185

19/413

6/35

4/96

1/8

14/259

UK-HARP-I 2005

0.99 (0.14, 7.10)

2/225

2/223

Middleton 1992

0.60 (0.35, 1.03)

23/451

37/452

0.01

0.1

1

5

10

Creek 1990

ELL 1982

0.98 (0.28, 3.46)

0.35 (0.01, 8.93)

5/144

0/24

5/141

1/26

**Study,Year**

**Treatment**

**Events/Patients**

**Control**

1042/13591

920/11117

**All-cause death**

**(I^2^ =0%, N=13)**

499/9702

444/8231

**Cardiovascular death**

**0.87 (0.71, 1.01)**

**0.91 (0.67, 1.13)**

CURE 2007

0.95 (0.78, 1.16)

196/2044

204/2043

**(I^2^ =0.8%, N=24)**

**Overall**

**Overall**

**Odds Radio (95% CI)**

Antiplatelet therapy better

Control better

CI = confidence interval; N = number of trials.
