# Supplementary material for: Effect of antiplatelet therapy on cardiovascular and kidney outcomes in patients with chronic kidney disease: a systematic review and meta-analysis
Source: BMC Nephrol. 2019 Aug 7;20:309. doi: 10.1186/s12882-019-1499-3 (PMC6686545; doi:10.1186/s12882-019-1499-3)
Supplement: Supplementary file 9 — Table S3. Subgroup Analysis of Outcomes and Adverse Events. (DOCX 20 kb) [file 12882_2019_1499_MOESM9_ESM.docx]

**Additional file 9: Table S3.** Subgroup Analysis of Outcomes and Adverse Events

Major cardiovascular events were defined as a composite including fatal or non-fatal myocardial infarction, fatal or non-fatal stroke, coronary artery revascularization, and cardiovascular death. Kidney failure events were defined as more than 25% or 50% decrease in eGFR, doubling of serum creatinine, or ESRD

| **Subgroups** | **Major cardiovascular events** | | **All-cause death** | | **Any Bleeding** | | **Access failure** | | **Kidney failure events** | | **Major Bleeding** | | **Minor Bleeding** | |  |
| --- | --- | --- | --- | --- | --- | --- | --- | --- | --- | --- | --- | --- | --- | --- | --- |
|  | **OR** | **P** | **OR** | **P** | **OR** | **P** | **OR** | **P** | **OR** | **P** | **OR** | **P** | **OR** | **P** | |
| **1. Clinical characteristics of participants** | | | | | | | | | | | | | | | |
| **1) CKD stage 5** | 0.71(0.52,0.99) | 0.2 | 0.80(0.63,1.02) | 0.4 | 1.30(1.00,1.68) | 0.09 | - | - |  | - | 1.16(0.83,1.63) | 0.3 | 1.36(0.91,2.03) | 0.09 | |
| **No-CKD stage 5** | 0.88(0.79,0.98) |  | 0.96(0.79,1.17) |  | 1.79(1.50,2.13) |  | - |  | - |  | 1.52(1.20,1.91) |  | 1.76(1.44,2.14) |  |  |
| **2) ACS/PCI populations** | 0.91(0.83,1.00) | 0.1 | 0.95(0.82,1.10) | 0.8 | 1.64(1.34,2.01) | 0.4 | - |  | - |  | 1.40(1.03,1.91) | 0.6 | 1.55(1.22,1.96) | 0.3 | |
| **Other populations** | 0.80(0.68,0.96) |  | 0.92(0.79,1.08) |  | 1.71(1.39,2.10) |  | - |  | - |  | 1.45(1.15,1.82) |  | 1.95(1.47,2.59) |  |  |
| **2. Drug types** | | | | | | | | | | | | | | | |
| **Cyclooxygenase-2 inhibitor** | 0.98(0.48,2.03) | 0.3 | 0.86(0.66,1.13) | 0.6 | 1.81(1.32,2.47) | 0.1 | 0.30(0.15,0.61) | **0.002** | 0.95(0.56,1.62) | 0.2 | 1.42(0.95,2.15) | 0.9 | 2.05(1.12,3.77) | 0.4 | |
| **Adenosine diphosphate P2Y12 receptor inhibitor** | 0.85(0.71,1.02) |  | 1.02(0.86,1.21) |  | - |  | 0.52(0.38,0.72) |  | 0.38(0.07,2.10) |  | 1.43(1.11,1.83) |  | 1.65(1.18,2.32) |  |  |
| **Platelet glycoprotein IIb/IIIa receptor blockade** | 0.91(0.81,1.01) |  | 0.78(0.54,1.15) |  | 1.59(1.20,2.09) |  | - |  | - |  | 1.47(0.93,2.32) |  | 1.80(1.46,2.22) |  |  |
| **Thromboxane A2 synthase and receptor** **inhibitor** | 0.45(0.19,1.05) |  | 0.93(0.47,1.81) |  | 1.73(1.43,2.11) |  | - |  | - |  | 1.04(0.26,4.18) |  | - |  |  |
| **Phosphodiesterase inhibitor** | - |  | - |  | 1.04(0.26,4.18) |  | - |  | 1.66(0.69,3.96) |  | - |  | - |  |  |
| **Combined therapy** | 0.65(0.15,2.88) |  | 0.79(0.60,1.03) |  | 1.79(0.87,3.73) |  | 0.87(0.69,1.11) |  | 0.29(0.06,1.33) |  | 1.39(0.78,2.48) |  | 1.53(0.68,3.41) |  |  |
| **3. Sample size** | | | | | | | | | | | | | | | |
| **<500** | 0.86(0.65,1.14) | 0.8 | 0.86(0.62,1.20) | 0.6 | 1.47(1.15,1.89) | 0.1 | 0.49(0.33,0.73) | 0.3 | - | 0.2 | 1.25(0.90,1.74) | 0.5 | 1.48(0.96,2.28) | 0.4 | |
| **≥500** | 0.85(0.76,0.96) |  | 0.93(0.80,1.08) |  | 1.79(1.52,2.10) |  | 0.72(0.47,1.11) |  | - |  | 1.45(1.17,1.79) |  | 1.80(1.50,2.16) |  |  |
| **4. Follow -up time** | | | | | | | | | | | | | | | |
| **<12 months** | 0.92(0.81,1.04) | 0.1 | 0.94(0.75,1.17) | 0.8 | 1.57(1.35,1.82) | 0.08 | 0.45(0.30,0.68) | 0.2 | - |  | 1.15(0.92,1.43) | 0.1 | 1.66(1.29,2.13) | 0.9 | |
| **≥12 months** | 0.84(0.72,0.98) |  | 0.93(0.82,1.07) |  | 1.82(1.50,2.20) |  | 0.87(0.67,1.13) |  | - |  | 1.61(1.29,2.01) |  | 1.81(1.42,2.31) |  |  |
| **5. Mean age** | | | | | | | | | | | | | | | |
| **<60 years** | 1.18(0.88,1.60) | 0.3 | 0.96(0.82,1.13) | 0.4 | 1.33(1.00,1.76) | 0.09 | 0.49(0.33,0.73) | 0.4 | 0.32(0.10,1.02) | 0.2 | 0.93(0.60,1.44) | 0.08 | 1.78(1.46,2.18) | 0.5 | |
| **≥60 years** | 0.89(0.78,0.98) |  | 0.89(0.66,1.19) |  | 1.76(1.52,2.05) |  | 0.93(0.51,1.68) |  | 0.95(0.56,1.62) |  | 1.55(1.26,1.90) |  | 1.62(1.05,2.49) |  |  |

ACS = cute coronary syndrome; CKD = chronic kidney disease; PCI = percutaneous coronary intervention; OR = odds radio.
