# Supplementary material for: Effect of antiplatelet therapy on cardiovascular and kidney outcomes in patients with chronic kidney disease: a systematic review and meta-analysis
Source: BMC Nephrol. 2019 Aug 7;20:309. doi: 10.1186/s12882-019-1499-3 (PMC6686545; doi:10.1186/s12882-019-1499-3)
Supplement: Supplementary file 10 — Figure S4. Forest plot for the rate of eGFR decline. (DOCX 54 kb) [file 12882_2019_1499_MOESM10_ESM.docx]

**Additional file 10: Figure S4. Forest plot for the rate of eGFR decline.**

Khajehdehi 2002

**Study, year**

HOT 2010(1)

HOT 2010(2)

Nyberg 1984

Luk 2010

Cheng 1998

Giustina 1998

Tang 2014

**0.15 (-0.89, 1.20)**

-0.60 (-90.04, 88.84)

-0.08 (-0.37, 0.21)

0.30 (-0.74, 1.34)

-6.00 (-15.85, 3.85)

-2.30 (-9.10, 4.50)

2.35 (-0.19, 4.89)

-30.00 (-53.91, -6.09)

-0.70 (-5.75, 4.35)

**Mean Difference (95% CI)**

0

-25

25

1527, NA

**Treatment**

57, -1.2 (33)

32, -5.1 (13.7)

45, -8.2 (12.7)

19, -6.52 (3.29)

15, -12 (33.4)

11, -9 (11.8)

1556, NA

19, -.6 (198)

30, -2.8 (13.7)

45, -7.5 (11.7)

12, -8.87 (3.65)

15, 18 (33.4)

11, -3 (11.8)

**N, mean (SD)**

**N, mean (SD)**

**Overall (I^2^ = 40.8%, *P* = 0.1)**

264, NA

276, NA

1970

1964

**Control**

Antiplatelet therapy better

Control better

Positive differences represent a slower decline in the antiplatelet treatment group than in the control group

CI = confidence interval; eGFR = estimated glomerular filtration rate; N = number of trials; NA = no available; SD = standard deviation.
