# Supplementary material for: Effect of antiplatelet therapy on cardiovascular and kidney outcomes in patients with chronic kidney disease: a systematic review and meta-analysis
Source: BMC Nephrol. 2019 Aug 7;20:309. doi: 10.1186/s12882-019-1499-3 (PMC6686545; doi:10.1186/s12882-019-1499-3)
Supplement: Supplementary file 11 — Figure S5. Forest plot for the change of serum creatinine. (DOCX 44 kb) [file 12882_2019_1499_MOESM11_ESM.docx]

**Additional file 11: Figure S5. Forest plot for the change of serum creatinine.**

**Overall (*I^2^*= 87.4%, *P* < 0.001)**

Khajehdehi 2002

Zauner 1994

Frascra 1996

Giustina 1998

**-7.92 (-30.41, 14.56)**

7.96 (-8.00, 23.92)

-15.00 (-50.17, 20.17)

-35.36 (-50.86, -19.86)

7.00 (-1.91, 15.91)

92

57, 1.77 (32.3)

10, -16 (41.5)

10, -22.1 (17.7)

15, -1.8 (8.8)

52

19, -6.19 (30.2)

8, -1 (34.6)

10, 13.3 (17.7)

15, -8.8 (15.2)

0

-50

-25

25

**Study, year**

**Mean Difference (95% CI)**

**Treatment**

**N, mean (SD)**

**N, mean (SD)**

**Control**

Antiplatelet therapy better

Control better

Negative differences represent a greater decrease in the treatment group than in the control group.

CI = confidence interval; N = number of trials; SD = standard deviation.
