# Supplementary material for: Effect of antiplatelet therapy on cardiovascular and kidney outcomes in patients with chronic kidney disease: a systematic review and meta-analysis
Source: BMC Nephrol. 2019 Aug 7;20:309. doi: 10.1186/s12882-019-1499-3 (PMC6686545; doi:10.1186/s12882-019-1499-3)
Supplement: Supplementary file 12 — Figure S6. Forest plot for the change of proteinuria or albuminuria. (DOCX 52 kb) [file 12882_2019_1499_MOESM12_ESM.docx]

**Additional file 12: Figure S6.** Forest plot for the change of proteinuria or albuminuria.

**Overall (*I^2^* = 70.0%, *P* = 0.001)**

Khajehdehi 2002

Frascra 1996

Zauner 1994

Jiao 2013

Giustina 1998

Cheng 1998

Tang 2014

Luk 2010

**-0.90 (-1.34, -0.47)**

-0.82 (-1.35, -0.28)

-1.27 (-2.24, -0.30)

**Standard Mean Difference (95% CI)**

-2.99 (-4.38, -1.59)

-1.36 (-2.05, -0.67)

-0.99 (-1.75, -0.23)

-0.62 (-1.36, 0.12)

-0.56 (-0.98, -0.14)

-0.03 (-0.52, 0.47)

**208**

57, -.28 (.37)

10, -.6 (.721)

10, -6.7 (1.21)

20, -56.2 (34.7)

15, -28 (34.1)

19, -82 (44)

45, -18.6 (255)

32, -31.9 (100)

**159**

19, .02 (.358)

10, .2 (.52)

8, -2.8 (1.42)

20, -4.18 (41.6)

15, 14 (49.5)

12, -36 (107)

45, 141 (311)

30, -29.4 (84.9)

0

-5

1

**Study, year**

**Treatment**

**N, mean (SD)**

**N, mean (SD)**

**Control**

Antiplatelet therapy better

Control better

Negative differences represent a greater decrease in the treatment group than in the control group.

CI = confidence interval; N = number of trials; SD = standard deviation.
