# Supplementary material for: Effect of antiplatelet therapy on cardiovascular and kidney outcomes in patients with chronic kidney disease: a systematic review and meta-analysis
Source: BMC Nephrol. 2019 Aug 7;20:309. doi: 10.1186/s12882-019-1499-3 (PMC6686545; doi:10.1186/s12882-019-1499-3)
Supplement: Supplementary file 13 — Figure S7. Forest plot for hemodialysis vascular access. (DOCX 67 kb) [file 12882_2019_1499_MOESM13_ESM.docx]

**Additional file 13: Figure S7.** Forest plot for hemodialysis vascular access.

Dember 2008

Kaegi 1974

Kooistra 1994

Ghorbani 2009

Dixon 2009

Abdul-Rahman 2007

Harter 1979

Andrassy 1974

Fiskerstrand 1985

Grontoft 1998

Sreedhara 1994

Grontoft 1985

Michie 1977

Rouzrokh 2010

**0.52 (0.31, 0.73)**

0.57 (0.39, 0.83)

0.17 (0.04, 0.63)

1.20 (0.35, 4.13)

0.22 (0.04, 1.11)

0.89 (0.65, 1.22)

0.30 (0.07, 1.23)

0.18 (0.05, 0.66)

0.15 (0.03, 0.73)

0.33 (0.04, 2.52)

0.60 (0.30, 1.19)

0.92 (0.37, 2.33)

0.13 (0.02, 0.76)

0.43 (0.03, 5.98)

0.83 (0.52, 1.32)

**369/1589**

53/435

12/24

6/69

2/46

127/321

4/19

6/19

2/45

2/8

16/129

33/83

2/19

1/8

70/260

**420/1409**

84/431

24/28

5/68

8/47

139/328

9/19

18/25

11/47

5/10

25/131

10/24

8/17

2/8

40/130

0.01

Kaufman 2003

0.93 (0.51, 1.68)

33/104

32/96

1

0.1

5

**Overall (*I^2^* = 45.7%, *P* = 0.03)**

**Odds Radio (95% CI)**

**Study,Year**

**Treatment**

**Events/Patients**

**Control**

Antiplatelet therapy better

Control better

CI = confidence interval.
