# Supplementary material for: Effect of antiplatelet therapy on cardiovascular and kidney outcomes in patients with chronic kidney disease: a systematic review and meta-analysis
Source: BMC Nephrol. 2019 Aug 7;20:309. doi: 10.1186/s12882-019-1499-3 (PMC6686545; doi:10.1186/s12882-019-1499-3)
Supplement: Supplementary file 14 — Figure S8. Forest plot for adverse events (major and minor Bleeding). (DOCX 61 kb) [file 12882_2019_1499_MOESM14_ESM.docx]

**Additional file 14: Figure S8.** Forest plot for adverse events (major and minor Bleeding).

CI = confidence interval; N = number of trials.
