# Supplementary material for: Effect of antiplatelet therapy on cardiovascular and kidney outcomes in patients with chronic kidney disease: a systematic review and meta-analysis
Source: BMC Nephrol. 2019 Aug 7;20:309. doi: 10.1186/s12882-019-1499-3 (PMC6686545; doi:10.1186/s12882-019-1499-3)
Supplement: Supplementary file 15 — Table S4. Sensitivity Analysis of Outcomes and Adverse Events. (DOCX 20 kb) [file 12882_2019_1499_MOESM15_ESM.docx]

**Additional file 15: Table S4.** Sensitivity Analysis of Outcomes and Adverse Events

CI = confidence interval; DL = DerSimonian-Laird; EB = empirical Bayes; FB = full Bayes; OR = odds ratio; REML = restricted maximum likelihood.

^*^ τ^2^ represents between-study heterogeneity characterized by standard deviation.

^†^ credible intervals of full Bayes estimator.

| **Sensitivity analysis** | **Major cardiovascular events** | | | **All-cause death** | | | **Access failure** | | | **Kidney failure events** | | | **Any bleeding** | | | **Major bleeding** | | | **Minor bleeding** | | |
| --- | --- | --- | --- | --- | --- | --- | --- | --- | --- | --- | --- | --- | --- | --- | --- | --- | --- | --- | --- | --- | --- |
|  | **n/N** | **OR** | **τ^2a^** | **n/N** | **OR** | **τ^2^** | **n/N** | **OR** | **τ^2^** | **n/N** | **OR** | **τ^2^** | **n/N** | **OR** | **τ^2^** | **n/N** | **OR** | **τ^2^** | **n/N** | **OR** | **τ^2^** |
| **Omit studies of follow-up time <12 months** | 16/  19741 | 0.83  (0.72,0.97) | 0.02 | 15/  16953 | 0.93  (0.82,1.07) | 0.01 | 4/  994 | 0.87  (0.67,1.13) | <0.0001 |  | - | - | 18  /20431 | 1.77  (1.42,2.24) | 0.08 | 15/  20100 | 1.61  (1.29,2.01) | 0.02 | 13/  17813 | 1.80  (1.42,2.30) | 0.08 |
| **Omit studies of sample size <200** | 19/  24807 | 0.86  (0.77,0.96) | 0.002 | 17/  20101 | 0.91  (0.76,1.09) | 0.006 | 6/  2365 | 0.76  (0.62,0.93) | 0.01 |  | - | - | 19/  25083 | 1.63  (1.33,1.99) | 0.08 | 19/  25189 | 1.39  (1.14,1.69) | 0.03 | 15/  22558 | 1.80  (1.38,2.35) | 0.06 |
| **Omit studies of Jadad score <3** | 20/  23882 | 0.87  (0.78,0.98) | 0.007 | 21/  23463 | 0.94  (0.79,1.11) | 0.003 | 11/  2713 | 0.61  (0.45,0.83) | 0.1 | 3/  673 | 0.82  (0.46,1.46) | 0.04 | 24/  23953 | 1.64  (1.37,1.96) | 0.07 | 23/  24016 | 1.32  (1.05,1.59) | 0.02 | 20/  22906 | 1.76  (1.39,2.23) | 0.01 |
| **Different statistical estimators** | | | | | | | | | | | | | | | | | | | | |  |
| **DL** | 25/  25315 | 0.88  (0.79,0.96) | 0.008 | 24/  24701 | 0.93  (0.85,1.03) | 0.005 | 15/  2998 | 0.58  (0.43,0.78) | 0.12 | 7/  811 | 0.90  (0.64,1.35) | 0.01 | 30/  26118 | 1.68  (1.47,1.94) | 0.04 | 27/  25928 | 1.38  (1.19,1.60) | 0.002 | 23/  23138 | 1.72  (1.44,2.05) | 0.06 |
| **EB** | 25/  25315 | 0.87  (0.78,0.97) | 0.01 | 24/  24701 | 0.93  (0.82,1.05) | 0.003 | 15/  2998 | 0.54  (0.37,0.79) | 0.21 | 7/  811 | 0.89  (0.50,1.38) | 0.02 | 30/  26118 | 1.68  (1.45,1.95) | 0.04 | 27/  25928 | 1.39  (1.16,1.65) | 0.007 | 23/  23138 | 1.72  (1.36,2.18) | 0.11 |
| **REML** | 25/  25315 | 0.87  (0.79,0.95) | 0.003 | 24/  24701 | 0.91  (0.77,1.07) | 0.03 | 15/  2998 | 0.56  (0.39,0.81) | 0.14 | 7/  811 | 0.90  (0.50,1.35) | <0.001 | 30/  26118 | 1.65  (1.40,1.96) | 0.07 | 27/  25928 | 1.39  (1.13,1.72) | 0.04 | 23/  23138 | 1.72  (1.37,2.17) | 0.1 |
| **FB^†^** | 25/  25315 | 0.84  (0.74,0.94) | 0.01 | 24/  24701 | 0.87  (0.71,1.01) | 0.05 | 15/  2998 | 0.52  (0.31,0.73) | 0.33 | 7/  811 | 0.87  (0.32,1.55) | 0.19 | 30/  26118 | 1.55  (1.25,1.84) | 0.09 | 27/  25928 | 1.33  (1.11,1.59) | 0.07 | 23/  23138 | 1.66  (1.27,2.05) | 0.15 |
