# Supplementary material for: Effect of antiplatelet therapy on cardiovascular and kidney outcomes in patients with chronic kidney disease: a systematic review and meta-analysis
Source: BMC Nephrol. 2019 Aug 7;20:309. doi: 10.1186/s12882-019-1499-3 (PMC6686545; doi:10.1186/s12882-019-1499-3)
Supplement: Supplementary file 16 — Table S5. Evidence Profile for the Effect of Antiplatelet Therapy on Outcomes in Patients With CKD. (DOCX 17 kb) [file 12882_2019_1499_MOESM16_ESM.docx]

**Additional file 16: Table S5. Evidence Profile for the Effect of Antiplatelet Therapy on Outcomes in Patients With CKD***

| **Outcome Variable** | **Participants (studies)** | **Quality assessment** | | | | | | **Summary of Findings** |
| --- | --- | --- | --- | --- | --- | --- | --- | --- |
|  |  | **Risk of bias** | **Inconsistency** | **Indirectness** | **Imprecision** | **Publication bias** | **Overall quality of evidence** | **OR/MD/SMD**  (95% CI) |
|  |  |  |  |  |  |  |  |  |
| **Cardiovascular events** | 25135 (25 studies) | serious | no serious inconsistency | serious | no serious imprecision | undetected | ⊕⊕⊝⊝ **LOW** | **0.85 (0.74, 0.94)** |
| **All-cause death** | 24708 (24 studies) | serious | no serious inconsistency | serious | no serious imprecision | undetected | ⊕⊕⊝⊝ **LOW** | 0.87 (0.71, 1.01) |
| **Access failure** | 2998 (15 studies) | serious | no serious inconsistency | serious | no serious imprecision | undetected | ⊕⊕⊝⊝ **LOW** | **0.52 (0.31, 0.73)** |
| **Kidney failure events** | 811 (6 studies) | serious | serious | serious | no serious imprecision | undetected | ⊕⊝⊝⊝ **VERY LOW** | 0.87 (0.32, 1.55) |
| **Major bleeding** | 25815 (27 studies) | serious | no serious inconsistency | serious | no serious imprecision | undetected | ⊕⊕⊝⊝ **LOW** | **1.33 (1.11, 1.59)** |
| **Minor bleeding** | 23138 (23 studies) | serious | no serious inconsistency | serious | no serious imprecision | undetected | ⊕⊕⊝⊝ **LOW** | **1.66 (1.27, 2.05)** |
| **Serum creatinine** | 144 (4 studies) | serious | no serious inconsistency | serious | serious | undetected | ⊕⊝⊝⊝ **VERY LOW** | 0.15 (-0.89, 1.20) |
| **eGFR** | 3934 (7 studies) | serious | no serious inconsistency | serious | serious | undetected | ⊕⊝⊝⊝ **VERY LOW** | -7.92 (-30.41, 14.56) |
| **Proteinuria or albuminuria** | 367 (8 studies) | serious | no serious inconsistency | serious | serious | undetected | ⊕⊝⊝⊝ **VERY LOW** | **-0.90 (-1.34, -0.47)** |

*Quality assessed according to the Grading of Recommendations Assessment, Development, and Evaluation guidelines.
