# Supplementary material for: Effect of antiplatelet therapy on cardiovascular and kidney outcomes in patients with chronic kidney disease: a systematic review and meta-analysis
Source: BMC Nephrol. 2019 Aug 7;20:309. doi: 10.1186/s12882-019-1499-3 (PMC6686545; doi:10.1186/s12882-019-1499-3)
Supplement: Supplementary file 17 — Figure S9. Funnel plots and Egger’s test for small study effects. (DOCX 29 kb) [file 12882_2019_1499_MOESM17_ESM.docx]

**Additional file 17: Fig S9. Funnel plots and Egger’s test for small study effects**

**A. Major cardiovascular events (*P* = 0.2) B. All-cause death (*P* = 0.1)**

**C. Access failure (*P* = 0.05) D. Kidney failure events (*P* = 0.6)**

**E. Major bleeding (*P* = 0.8) F. Minor bleeding (*P* = 0.4)**
